# Treatment‐emergent central sleep apnoea managed by CPAP with adjunctive acetazolamide: A case report

**DOI:** 10.1002/rcr2.916

**Published:** 2022-02-27

**Authors:** Chin Tong Kwok, Kam Cheung Wong, Chun Lee Kwok, Sing Hang Lee, Kwok Sang Yee

**Affiliations:** ^1^ Department of Tuberculosis and Chest Tung Wah Group of Hospitals Wong Tai Sin Hospital Hong Kong China

**Keywords:** acetazolamide, continuous positive airway pressure‐emergent central sleep apnoea, loop gain, obstructive sleep apnoea, treatment‐emergent central sleep apnoea

## Abstract

Treatment‐emergent central sleep apnoea (TECSA) refers to the emergence of central apnoea during treatment for obstructive sleep apnoea (OSA), most commonly continuous positive airway pressure (CPAP). It has been reported in 8% of OSA patients treated with CPAP and spontaneous resolution rate varies between 60% and 80%. Management options include watchful waiting with continuation of CPAP, bi‐level positive pressure ventilation, adaptive servo‐ventilation and CPAP with supplemental oxygen. Acetazolamide has been shown to be effective in other forms of central sleep apnoea; its use as adjunct to CPAP in TECSA is sparsely reported. We report a 74‐year‐old man with severe OSA who developed moderate central apnoea upon CPAP initiation. Subsequent addition of acetazolamide led to gratifying resolution of the TECSA. In TECSA patients with significant symptoms and high central apnoea index, treatment with acetazolamide as adjunct to CPAP may be considered, particularly in patients in whom CPAP adherence is imperative.

## Graphical abstract

We report a 74‐year‐old man with severe obstructive sleep apnoea who developed moderate central apnoea upon continuous positive airway pressure (CPAP) initiation. Subsequent addition of acetazolamide led to gratifying resolution of the treatment‐emergent central sleep apnoea (TECSA). In TECSA patients with significant symptoms and high central apnoea index, treatment with acetazolamide as adjunct to CPAP may be considered, particularly in patients in whom CPAP adherence is imperative.

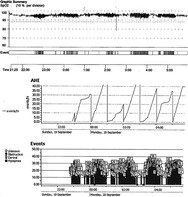

## INTRODUCTION

We report a severe obstructive sleep apnoea (OSA) patient with multiple co‐morbidities, who developed treatment‐emergent central sleep apnoea (TECSA) upon continuous positive airway pressure (CPAP) initiation. The significant symptoms and high central apnoea index (CAI) warranted an empirical trial of acetazolamide as adjunct to CPAP.

## CASE REPORT

A 74‐year‐old Chinese man presented to our sleep clinic with a 5‐year history of loud snoring and excessive daytime sleepiness. His score of Epworth sleepiness scale (ESS) was 17. He was a construction site worker; he never smokes nor consumes alcohol. He had multiple co‐morbid conditions, including hypertension, hyperlipidaemia, atrial fibrillation (AF), diastolic heart failure (by echocardiogram), transient ischaemic attack, anxiety and insomnia. Physical examination revealed an obese man with a body mass index (BMI) of 29.5, irregular pulse consistent with slow AF (subsequently confirmed by electrocardiogram) and there was no sign of heart failure.

Polysomnography (PSG) manually scored according to the American Academy of Sleep Medicine (AASM) scoring rules revealed: total sleep time 395 min; and apnoea–hypopnoea index (AHI), CAI and oxygen desaturation index (ODI) were 40.3, 2.3 and 38.1/h, respectively. Severe OSA (hypopnoea predominant) was diagnosed. Details of PSG results are summarized in Table 1.

**TABLE 1:** The PSG showing severe obstructive sleep apnoea (hypopnoea predominant)

Total sleep time	395 min
Sleep efficiency	TST/TRT 71.1%
AHI	40.3/h
AHI during REM sleep^a^	29.6/h
AHI during NREM sleep^a^	39.0/h
Central apnoea index	2.3/h
Mixed apnoea index	2.1/h
Obstructive apnoea index	5.5/h
Hypopnoea index	30.4/h
ODI	38.1/h
Lowest oxygen saturation (SpO_2_)	86%
Total arousal index (spontaneous)^a^	22.9/h (9.7/h)

Abbreviations: AHI, apnoea–hypopnoea index; NREM, non‐REM; ODI, oxygen desaturation index; PSG, polysomnography; REM, rapid eye movement; TST/TRT, Total sleep time/Total recording time.

^a^ The AHI during NREM sleep was higher than AHI during REM sleep. The total arousal index was high.

**FIGURE 1:**
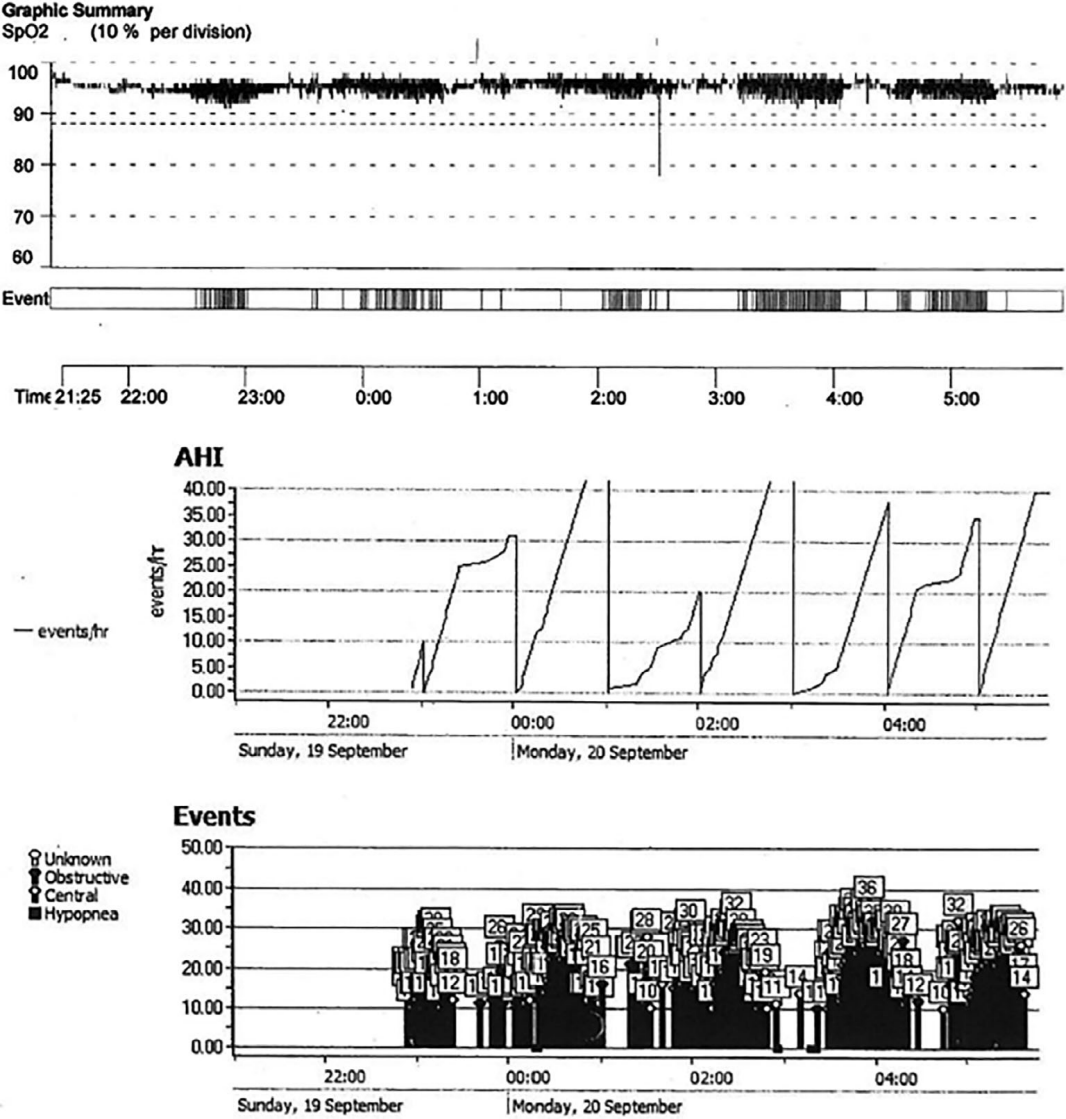
The auto‐continuous positive airway pressure titration using S9 AutoSet device illustrates that the respiratory events (apnoea–hypopnoea index [AHI] and individual events in middle and bottom figures) largely correlated temporally with desaturation on pulse oximetry (in the upper figure) (AHI, central apnoea index and oxygen desaturation index were 32.8, 26.6 and 19.8/h, respectively)

Auto‐CPAP titration using S9 AutoSet (ResMed) device was then performed in our sleep laboratory. The machine‐measured AHI was 32.8/h with a predominance of central apnoea and CAI was 26.6/h, while ODI was 19.8/h. The respiratory events correlated temporally with desaturation on pulse oximetry (Figure 1). Central apnoea of similar severity persisted in several CPAP trials of fixed pressure ranging from 10 to 12 cm H_2_O in our sleep laboratory. Symptomatically, there was little improvement with an ESS score of 16. The diagnosis of CPAP‐emergent central sleep apnoea was made.

The patient was prescribed oral acetazolamide of 125 mg nocte as adjunct to fixed nasal CPAP 10 cm H_2_O as empirical treatment of TECSA, after repeat auto‐CPAP titration with adjunctive acetazolamide. He reported satisfactory tolerance of CPAP and acetazolamide, waking up refreshed and resolution of daytime sleepiness. His ESS score was diminished to 10. Data from fixed CPAP 10 cm H_2_O with adjunctive acetazolamide treatment for 4 weeks showed dramatic resolution of CAI to 0.3/h, while AHI and ODI were 1.8 and 0.3/h, respectively. The TECSA was successfully treated by acetazolamide as adjunct to CPAP.

Subsequently, the patient was instructed to suspend acetazolamide for 1 week as a trial while continuing CPAP. Patient reported sleep fragmentation waking up unrefreshed. Data from CPAP 10 cm H_2_O revealed re‐emergence of TECSA with CAI of 20.5/h, while AHI and ODI were 26.2 and 10.1/h, respectively. Table 2 shows a summary of the clinical and physiological parameters with and without adjunctive acetazolamide.

**TABLE 2:** Summary of ESS score and physiological parameters upon PAP treatment with and without adjunctive acetazolamide

	PSG	Auto‐CPAP titration without acetazolamide	CPAP with adjunctive acetazolamide	CPAP upon suspension of acetazolamide
ESS	17	16	10	16
AHI	40.3	32.8	1.8	26.2
CAI	2.3	26.6	0.3	20.5
ODI	38.1	19.8	0.3	10.1

Abbreviations: AHI, apnoea–hypopnoea index; CAI, central apnoea index; CPAP, continuous positive airway pressure; ESS, Epworth sleepiness scale; ODI, oxygen desaturation index; PSG, polysomnography.

The patient was advised to continue CPAP and adjunctive acetazolamide. A repeat suspension of adjunctive acetazolamide is planned 6 months later to elucidate whether TECSA will persist.

## DISCUSSION

In the International Classification of Sleep Disorders – third edition, TECSA refers to the emergence of central apnoea upon PAP treatment for OSA with CAI ≥5/h and ≥50% of events being central apnoea; and the central sleep apnoea (CSA) is not otherwise better explained. TECSA also occurs with other treatment modality for OSA including mandibular advancement device, maxillofacial surgery and tracheostomy. The prevalence is 8% among OSA patients upon CPAP titration.^1^ Risk factors include male gender, old age, lower BMI, heart failure, AF, high AHI, high CAI, high arousal index and high CPAP setting.^1^, ^2^

The mechanisms of central sleep apnoea encompass high loop gain, low arousal threshold, activation of lung stretch receptor and prolonged circulation time.^2^ Loop gain consisting of controller gain and plant gain is the ventilatory response to disturbance. Controller gain is the chemo‐responsiveness to change in partial pressure of carbon dioxide (PaCO_2_) level. Plant gain is the effectiveness of ventilation to eliminate CO_2_. In low arousal threshold state, frequent transition between arousal and sleep predisposes to ventilatory instability. Lung over‐stretching, when CPAP is over‐titrated, inhibits ventilation via vagal reflex.^2^ Prolonged circulation time results in a mismatch of PaCO_2_ with chemoreceptors, thus delaying ventilatory response.

The postulated pathophysiological factors of TECSA include high loop gain, intermittent decrease in carbon dioxide level below apnoeic threshold with CPAP and frequent arousals related to impaired sleep quality inducible by CPAP initiation.

Treatment options for TECSA^3^ include, first, expectant treatment with CPAP for patients with minimal TECSA symptoms and AHI below 15. High CAI and TECSA symptoms are known risk factors for poor CPAP adherence^4^; expectant treatment may not be advisable in those patients, especially when CPAP adherence is imperative. Other options^3^ were bi‐level positive airway pressure with back‐up rate, adaptive servo‐ventilation, CPAP with supplemental oxygen or adjunctive acetazolamide. In addition, non‐supine sleep posture by reducing loop gain is a possible treatment for TECSA.^5^

Acetazolamide is effective in treating idiopathic CSA, high‐altitude periodic breathing, CSA with heart failure and opioid‐induced CSA.^6^ Acetazolamide has also been successfully used as adjunct to CPAP in treating TECSA in whom CPAP with supplemental oxygen has failed.^6^

The demographic and clinical risk factors that our patient have for TECSA include male, old age, AF and diastolic heart failure, whereas high arousal index and higher AHI (non‐rapid eye movement) versus AHI (rapid eye movement) are pre‐disposing PSG parameters. The presence of significant TECSA symptoms and high CAI with corresponding ODI upon CPAP treatment warranted a trial of acetazolamide as adjunct. The re‐emergence of TECSA upon suspending acetazolamide was further evidence of its therapeutic effect as adjunct to CPAP in our patient. Further studies are warranted to elucidate the role of adjunctive acetazolamide to CPAP in the treatment of TECSA.

## CONFLICT OF INTEREST

None declared.

## AUTHOR CONTRIBUTION

Chin Tong Kwok drafted the manuscript. Kam Cheung Wong critically revised the manuscript. All authors have contributed substantially to the patient care and approved the final version.

## ETHICS STATEMENT

The authors declare that appropriate written informed consent was obtained for the publication of this manuscript and accompanying images.

## Data Availability

The data that support the findings of this study are available on request from the corresponding author. The data are not publicly available due to privacy or ethical restrictions.

## References

[1] Nigam G , Pathak C , Riaz M . A systematic review on prevalence and risk factors associated with treatment‐emergent central sleep apnea. Ann Thorac Med. 2016;11(3):202–10. 27512510 10.4103/1817-1737.185761 PMC4966223

[2] Zhang J , Wang L , Guo HJ , Wang Y , Cao J , Chen BY . Treatment‐emergent central sleep apnea: a unique sleep‐disordered breathing. Chin Med J (Engl). 2020;133(22):2721–30. 33009018 10.1097/CM9.0000000000001125 PMC7725531

[3] Zeineddine S , Badr MS . Treatment‐emergent central apnea: physiologic mechanisms informing clinical practice. Chest. 2021;159(6):2449–57. 33497650 10.1016/j.chest.2021.01.036 PMC8411449

[4] Mulgrew AT , Lawati NA , Ayas NT , Fox N , Hamilton P , Cortes L , et al. Residual sleep apnea on polysomnography after 3 months of CPAP therapy: clinical implications, predictors and patterns. Sleep Med. 2010;11(2):119–25. 20083429 10.1016/j.sleep.2009.05.017

[5] Joosten SA, Landry SA, Sands SA, Terrill PI, Mann D, et al. Dynamic loop gain increases upon adopting the supine body position during sleep in patients with obstructive sleep apnoea. Respirology. 2017;22(8):1662–9. 28730724 10.1111/resp.13108 PMC5895090

[6] Glidewell RN , Orr WC , Imes N . Acetazolamide as an adjunct to CPAP treatment: a case of complex sleep apnea in a patient on long‐acting opioid therapy. J Clin Sleep Med. 2009;5(1):63–4. 19317383 PMC2637168

